# Acute effects of traditional Japanese alcohol beverages on blood glucose and polysomnography levels in healthy subjects

**DOI:** 10.7717/peerj.1853

**Published:** 2016-04-04

**Authors:** Megumi Kido, Akihiro Asakawa, Ken-Ichiro K. Koyama, Toshio Takaoka, Aya Tajima, Shigeru Takaoka, Yumiko Yoshizaki, Kayu Okutsu, Kazunori T. Takamine, Yoshihiro Sameshima, Akio Inui

**Affiliations:** 1 Psychosomatic Internal Medicine, Kagoshima University Graduate School of Medical and Dental Sciences, Kagoshima, Japan; 2 The Japanese Society of Sleep Research, Kagoshima Takaoka Hospital, Kagoshima, Japan; 3 Kagoshima Takaoka Hospital, Kagoshima, Japan; 4 Laboratory of Shochu Fermentation Technology, Faculty of Agriculture, Kagoshima University, Kagoshima, Japan

**Keywords:** *Sake*, *Shochu*, Alcohol, Postprandial glucose levels, Insulin levels, Tipsiness, *Koji*

## Abstract

**Background.** Alcohol consumption is a lifestyle factor associated with type 2 diabetes. This relationship is reportedly different depending on the type of alcohol beverage. The purpose of this study was to examine the acute effects of traditional Japanese alcohol beverages on biochemical parameters, physical and emotional state, and sleep patterns.

**Methods.** Six healthy subjects (three men and three women; age, 28.8 ± 9.5 years; body mass index, 21.4 ± 1.6 kg/m^2^) consumed three different types of alcohol beverages (beer, *shochu*, and *sake*, each with 40 g ethanol) or mineral water with dinner on different days in the hospital. Blood samples were collected before and 1, 2, and 12 h after drinking each beverage, and assessments of physical and emotional state were administered at the same time. In addition, sleep patterns and brain waves were examined using polysomnography.

**Results.** Blood glucose levels at 1 h and the 12-h area under the curve (AUC) value after drinking *shochu* were significantly lower than that with water and beer. The 12-h blood insulin AUC value after drinking *shochu* was significantly lower than that with beer. Blood glucose × insulin level at 1 h and the 2-h blood glucose × insulin AUC value with *shochu* were significantly lower than that with beer. The insulinogenic indexes at 2 h with beer and *sake*, but not *shochu*, were significantly higher than that with water. The visual analogue scale scores of physical and emotional state showed that the tipsiness levels with beer, *shochu*, and *sake* at 1 h were significantly higher than that with water. These tipsiness levels were maintained at 2 h. The polysomnography showed that the rapid eye movement (REM) sleep latency with *shochu* and *sake* were shorter than that with water and beer.

**Conclusions.** Acute consumption of alcohol beverages with a meal resulted in different responses in postprandial glucose and insulin levels as well as REM sleep latency. Alcohol beverage type should be taken into consideration for people with impaired glucose tolerance.

## Background

Rates of type 2 diabetes have greatly increased over the past 50 years, primarily because of lifestyles changes, lack of physical activity, and high energy intake leading to overweight and obesity (Shaw, Sicree & Zimmet, 2010). The global prevalence of diabetes is expected to grow 69% over the next 20 years, increasing from 285 million in 2010 to 439 million in 2030 (Shaw, Sicree & Zimmet, 2010).

Alcohol consumption is a part of the culture and daily life worldwide, and plays an essential role in business and social activities (Coltart et al., 2011). By contrast, alcohol consumption is a lifestyle factor associated with type 2 diabetes. A large epidemiological study has reported that moderate alcohol intake over many years improves glucose tolerance and reduces the glucose induced insulin secretion, implying increased insulin sensitivity (Facchini, Chen & Reaven, 1994). In contrast, heavy drinkers results in impaired glucose tolerance (Shah, 1988). Several studies have reported a J-shaped relationship between alcohol consumption and insulin sensitivity and plasma insulin concentrations. (Hodge et al., 2006; Baliunas et al., 2009)

There are many different types of alcohol in the world, which has the unique flavor, taste and functional components. Alcohol metabolism is reportedly different depending on the type of alcohol beverage, and involvement of functional components was reported (Athyros et al., 2008; Carmen et al., 2011). It has been reported that resveratrol, the polyphenols contained in red wine, possesses cardioprotective effects, anti-inflammatory and anticancer properties (Hung et al., 2000; Baur & Sinclair, 2006). Additionally, resveratrol inhibits the oxidation of LDL cholesterol and prevent arteriosclerosis (Frankel, Waterhouse & Kinsella, 1993). Besides, isohumulones, bitter components derived from beer hops, is activates the peroxisome proliferator-activated receptor, and improve insulin sensitivity and lipid metabolism (Yajima et al., 2004; Miura et al., 2005).

*Shochu* and *sake* are traditional alcohol beverages in Japan. *Shochu* is made from barley, rice, sweet potato, sugar cane, or buckwheat. The *shochu* production process involves saccharification of starch through fermentation by *koji* and yeast, followed by distillation. Production of *shochu* differs from that of brandy and whiskey because it does not involve use of fruits or cereal germination as raw material. It has been reported that after consumption of *shochu*, plasma levels of urokinase-like plasminogen activator reportedly increase, and which decreases coronary heart disease and stroke risk (Sumi et al., 1988). *Sake* is a brewed beverage, which is called Japanese rice wine. It is prepared from steamed rice using fermentations by *koji* and yeast. The components of *sake*, such as ethyl *α*-d-glucoside and peptide fraction, Val—Tyr, His—Tyr, Arg—Phe, Val—Trp, and Tyr—Trp, have hepatoprotective actions and hypotensive effects (Saito et al., 1994; Izu et al., 2007).

However, little is known about how different types of traditional alcohol beverages affect metabolism and the physical and emotional state. Therefore, we investigated the acute effects of alcohol beverages consumed with a meal on biochemical parameters, physical and emotional state, sleep patterns, and brain waves. It is important in the pathogenesis of various diseases to clarify the acute effect on the metabolism of different types of alcohol.

## Methods

Six healthy subjects (three men and three women; age, 28.8 ± 9.5 years; body mass index, 21.4 ± 1.6 kg/m^2^) participated in this study. They had no remarkable medical history. Informed consent was obtained from each subject, and this study protocol was approved by the Takaoka Hospital ethics committee (Committee number 2009–001). The subjects participated once a week for four weeks, remaining in the hospital overnight at each visit. They were required to rest and not to eat after lunch until the experiment begins. Four beverages were randomly provided to drink. The beverages were beer (Budweiser, Kirin Brewery Co. Ltd., Tokyo, Japan); *shochu* (Shiranami, Satsuma-Shuzo Co. Ltd., Kagoshima, Japan), which is made from sweet potato; and *sake* (Yamadanishiki, Hakutsuru *Sake* Brewing Co. Ltd., Hyogo, Japan); and mineral water (Deeside, Whisk-e, Co. Ltd., Tokyo, Japan).

The six subjects were instructed to drink beer (1,000 ml, 5 % alcohol, 40 g alcohol, 400 kcal), *shochu* (333 ml, 15% alcohol, 40 g alcohol, 280 kcal, diluted with mineral water to a volume equal to the beer), *sake* (333 ml, 15% alcohol, 40 g alcohol, 329 kcal, diluted with mineral water to a volume equal to the beer), or mineral water (0 kcal, 1,000 ml, as a control) on the different days. Each beverage was provided at room temperature. The beverages drunk with dinner meal at 7:00 p.m. were consumed in 30 min. The dinner from the hospital menu for inpatients was used (energy, 709.6 ± 7.8 kcal; energy from carbohydrates, 448.0 ± 8.4 kcal; energy from protein, 106.7 ± 4.4 kcal; and energy from fat, 154.9 ± 9.7 kcal).

Blood samples were collected before consumption of the beverages and 1, 2, and 12 h after finishing the beverages. The effects of the alcohol beverages on physical and emotional state were assessed using visual analogue scales (VAS) at the same time. In this study, the VAS ranged from 0 to 100 mm. These scales require that the subject places a mark along a 100 mm vertical line to indicate how the subject presently feels. The VAS items asked if the subjects experienced headache, palpitations, appetite (desire for food), hunger (physical need for food), happiness, fullness, tipsiness (slight intoxication), weariness (tiredness or exhaustion), alcohol-related sickness, a feeling of relaxation, or nausea.

The effects of the alcohol beverages on sleep patterns and brain waves were examined using polysomnography (PSG). Lights were turned off at 9:00 p.m. and turned on again at 7:00 a.m. The PSG recorded brain waves (electroencephalogram), eye movements (electrooculogram), muscle activity (electromyogram), heart rhythm (electrocardiogram), and breathing during sleep.

All biochemical parameters (alcohol, glucose, insulin, prothrombin time, activated partial thromboplastin time, thrombo test, hepaplastin test, platelet aggregation activity, and lactic acid) were determined by the Clinical Pathology Laboratory, Ltd. (Kagoshima, Japan). Alcohol and insulin were measured using gas chromatography and chemiluminescence immunoassay, respectively. Glucose, ketone body fraction, and lactic acid were measured using an enzyme method. Prothrombin time, activated partial thromboplastin time, the thrombo test, and the hepaplastin test were measured using a light scattering method. Platelet aggregation (adenosine diphosphate and collagen) was measured using light transmission.

### Date treatment and statistical analysis

The blood glucose and insulin ratio was calculated using the following equation: blood glucose (mg/dl)/blood insulin (µU/ml). The insulinogenic index was calculated as the ratio of the increment of blood insulin to that of blood glucose after drinking: [blood insulin after drinking—fasting blood insulin (µU/ml)]/(blood glucose after drinking—fasting blood glucose (mg/dl)). Cumulative changes in blood alcohol, glucose, insulin responses, glucose-to-insulin ratio, and insulinogenic index were quantified as the incremental area under the curve (AUC) and were calculated using the trapezoidal rule with fasting concentrations representing the baseline truncated at zero. Any area below the baseline was ignored.

Statistical analyses were performed using SPSS software, and data are presented as mean ± standard error. Blood samples and the VAS scores were analyzed using two-way repeated measures analyses of variance (ANOVA), followed by post hoc analysis using the least significant difference (LSD) method. AUC values and sleep variables were analyzed using one-way repeated measures ANOVA, followed by post hoc analysis using the LSD method. All results were considered significant at *p* < 0.05.

## Results

From the six subjects, one woman did not completely drink the three types of alcohol and was excluded from the analysis. Because this subject is weak drinker.

**Figure 1:**
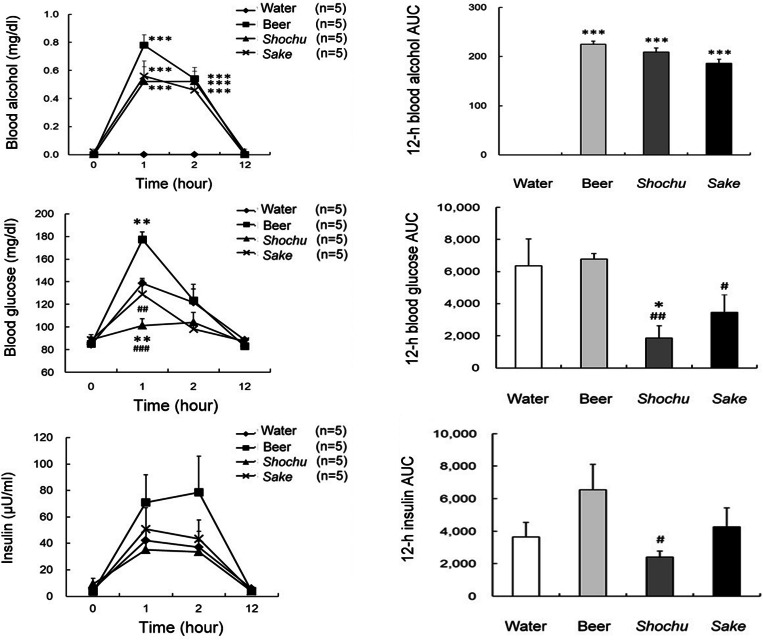
Blood glucose and insulin levels after drinking four types of beverages in healthy subjects. Blood alcohol levels (A1), the 12-h area under the curve (AUC) values for blood alcohol (A2), blood glucose levels (B1), the 12-h blood glucose AUC values (B2), blood insulin levels (C1), and the 12-h blood insulin AUC values (C2) after drinking four types of beverages in healthy subjects. Data are expressed as mean ± standard error (*n* = 5). Two-way repeated measures analysis of variance and post hoc analysis using least significant differences were used to compare clinical data between the beverages. ^∗^*p* < 0.05, ^∗∗^*p* < 0.01, ^∗∗∗^*p* < 0.001, compared with water; #*p* < 0.05, ##*p* < 0.01, ###*p* < 0.001, compared with beer.

**Figure 2:**
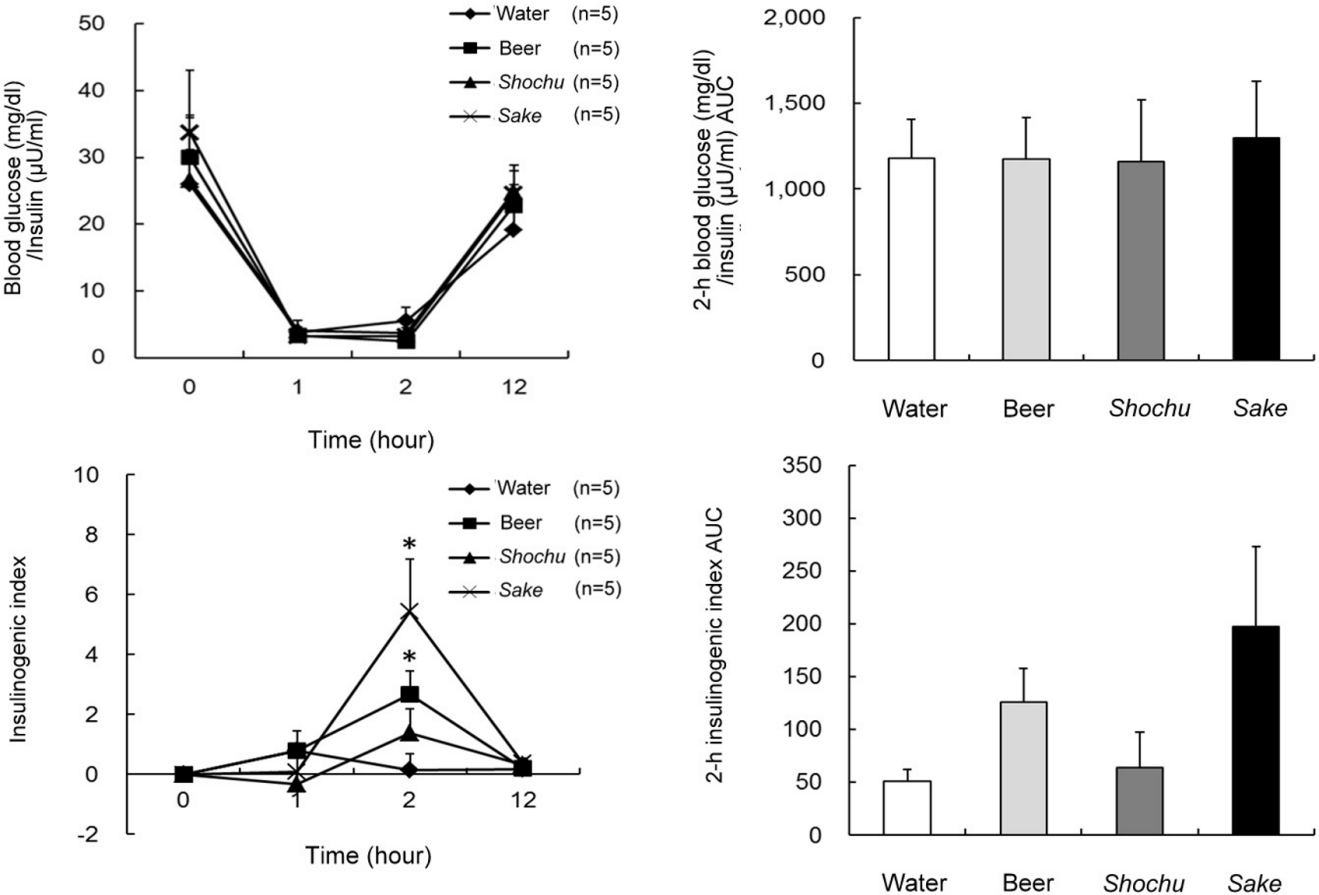
Blood glucose and insulin ratio levels, and insulinogenic index after drinking four types of beverages in healthy subjects. Blood glucose and insulin ratio levels (A1), the 2-h area under the curve (AUC) values for blood glucose and insulin ratio (A2), insulinogenic index (B1), and the 2-h AUC values for insulinogenic index (B2) after drinking four types of beverages in healthy subjects. Data are expressed as mean ± standard error (*n* = 5). Two-way repeated measures analysis of variance and post hoc analysis using least significant differences were used to compare clinical data between the beverages. ^∗^*p* < 0.05, compared with water.

The effects on the biochemical parameters were investigated by alcohol beverage type. The blood alcohol levels after consumption of beer, *shochu*, and *sake* at 1 and 2 h were significantly higher than that after consumption of water (*p* < 0.001 at both 1 and 2 h, Fig. 1). The 12-h blood alcohol AUC values with beer, *shochu*, and *sake* were significantly higher than that with water (*p* < 0.001). *Shochu* resulted in the lowest blood glucose level at 1 h, which was significantly lower than that with water and beer (*p* < 0.01). In contrast, beer resulted in a significantly higher blood glucose level at 1 h compared with the other groups (*p* < 0.01). The 12-h blood glucose AUC value with *shochu* was significantly lower than that with water and beer (*p* < 0.01), and the 12-h blood glucose AUC value with *sake* was significantly lower than that with beer (*p* < 0.05). Blood insulin levels were also the lowest with *shochu*. In addition, the 12-h blood insulin AUC value with *shochu* was significantly lower than that with beer (*p* < 0.05). Blood glucose levels, insulin levels, and the corresponding 12-h AUC values were not significantly different between *shochu* and *sake*. The blood glucose-to-insulin ratio and the corresponding 2-h AUC values were not significantly different between the beverages (Fig. 2). However, the insulinogenic indexes at 2 h with beer and *sake*, but not *shochu*, were significantly higher than that with water (*p* < 0.05).

The total keton levels with beer, *shochu*, and *sake* at 1 and 2 h were significantly higher than that with water (all, *p* < 0.01 at 1 h; *p* < 0.05, *p* < 0.01, and *p* < 0.05, respectively, at 2 h; Table 1). The total ketone levels before *shochu* and *sake* intake were significantly higher than that with beer (*p* < 0.05). The acetoacetate levels with beer and *sake* at 2 h were significantly lower than that with water (*p* < 0.05). The acetoacetate level before *shochu* intake was significantly lower than that with beer (*p* < 0.05). The acetoacetate level with *sake* at 2 h was significantly lower than that with *shochu* (*p* < 0.05). The 3-hydroxybutyric acid levels with beer, *shochu*, and *sake* at 1 and 2 h were significantly higher than that with water (all, *p* < 0.01 at 1 h; *p* < 0.05, *p* < 0.01, and *p* < 0.01, respectively, at 2 h). The 3-hydroxybutyric acid levels before *shochu* and *sake* intake were significantly higher than that with beer (*p* < 0.01). The lactic acid levels with beer, *shochu*, and *sake* at 2 were significantly higher than that with water (*p* < 0.01, *p* < 0.01, and *p* < 0.05, respectively). Prothrombin time, activated partial thromboplastin time, the hepaplastin test, collagen, and platelet aggregation activity levels were not significantly different between the beverages. The total ketone, acetoacetate, and 3-hydroxybutyric acid levels were variable before intake of alcohol beverages, probably reflecting feeding and activity status on that day since the experiments were performed at night, the time usually enjoying alcohol beverages.

**Table 1:** Measurement levels before and after drinking four types of beverages in healthy subjects. Data are expressed as mean ± standard error (*n* = 5). Two-way repeated measures analysis of variance and post hoc analysis using least significant differences were used to compare clinical data between the beverages. ^∗^*p* < 0.05, ^∗∗^*p* < 0.01, compared with water; #*p* < 0.05, ##*p* < 0.01, compared with beer; †*p* < 0.05, compared with *shochu*.

	Before intake	After 1 h	After 2 h	After 12 h
Total ketone (μmol/l)				
Water	104.82 ± 55.10	29.80 ± 2.67	30.02 ± 3.36	43.80 ± 14.98
Beer	160.72 ± 31.65	80.18 ± 7.09**	56.14 ± 10.76*	30.00 ± 4.57
*Shochu*	51.30 ± 12.44^#^	64.54 ± 7.70**	83.86 ± 11.50**	35.26 ± 9.26
*Sake*	54.20 ± 11.35^#^	67.50 ± 8.50**	58.00 ± 9.76*	29.56 ± 3.93
Acetoacetate (μmol/l)				
Water	28.58 ± 8.89	12.70 ± 0.85	17.90 ± 2.17	15.14 ± 2.80
Beer	40.60 ± 7.92	14.90 ± 2.88	11.84 ± 0.71*	13.46 ± 1.53
*Shochu*	20.76 ± 4.39	13.48 ± 1.50	15.32 ± 1.50	14.96 ± 3.05
*Sake*	19.82 ± 3.33^#^	11.82 ± 1.20	10.82 ± 0.53*^†^	11.56 ± 0.90
3-hydroxybutyric acid (μmol/l)				
Water	76.24 ± 46.28	17.08 ± 2.61	12.72 ± 1.41	29.92 ± 11.77
Beer	120.12 ± 24.02	55.36 ± 7.40**	44.64 ± 11.42*	17.48 ± 2.62
*Shochu*	31.16 ± 9.71^##^	52.92 ± 6.53**	68.36 ± 12.03**	21.00 ± 5.92
*Sake*	34.38 ± 8.36^##^	56.24 ± 7.84**	48.04 ± 9.36**	18.96 ± 2.58
Lactic acid (mg/dl)				
Water	7.98 ± 0.95	14.36 ± 1.78	11.16 ± 1.50	7.54 ± 0.85
Beer	6.32 ± 0.96	15.44 ± 2.07	18.32 ± 1.22**	8.98 ± 0.95
*Shochu*	8.20 ± 1.73	16.86 ± 1.77	18.66 ± 1.50**	7.38 ± 0.48
*Sake*	7.50 ± 0.70	16.54 ± 1.36	17.20 ± 2.01*	7.78 ± 0.56
Prothrombin time (sec)				
Water	11.78 ± 0.19	12.00 ± 0.35	11.86 ± 0.37	11.92 ± 0.38
Beer	11.58 ± 0.24	11.82 ± 0.26	11.50 ± 0.31	11.66 ± 0.23
*Shochu*	11.82 ± 0.31	11.78 ± 0.30	11.78 ± 0.30	11.86 ± 0.37
*Sake*	11.70 ± 0.37	11.86 ± 0.38	11.66 ± 0.32	11.74 ± 0.33
Activated partial thromboplastin time (sec)				
Water	26.72 ± 1.67	27.58 ± 2.02	26.58 ± 2.02	27.10 ± 2.00
Beer	26.04 ± 1.36	27.42 ± 1.89	25.96 ± 1.58	25.86 ± 1.29
*Shochu*	26.98 ± 1.81	27.26 ± 1.46	26.72 ± 1.54	26.98 ± 1.76
*Sake*	26.20 ± 1.49	27.02 ± 1.64	26.40 ± 1.48	26.42 ± 1.51
Thrombo test (%)				
Water	95.40 ± 4.60	94.00 ± 6.00	93.00 ± 3.77	85.00 ± 8.58
Beer	93.00 ± 3.77	90.00 ± 5.17	95.40 ± 4.60	90.20 ± 3.79
*Shochu*	95.80 ± 2.24	93.60 ± 3.93	98.40 ± 1.17	91.40 ± 3.41
*Sake*	93.40 ± 6.60	93.80 ± 6.20	94.60 ± 5.40	92.00 ± 6.20
Hepaplastin test (%)				
Water	105.80 ± 3.75	96.80 ± 5.54	99.20 ± 7.19	92.00 ± 6.20
Beer	105.60 ± 4.86	97.00 ± 5.73	105.00 ± 6.69	98.20 ± 3.89
*Shochu*	99.60 ± 4.89	98.00 ± 4.49	98.40 ± 4.50	93.60 ± 4.13
*Sake*	105.80 ± 6.72	103.00 ± 6.40	106.60 ± 7.05	98.60 ± 5.71
Adenosine diphosphate induced platelet aggregation (%)				
Water	88.60 ± 3.71	97.20 ± 5.45	88.80 ± 5.76	88.40 ± 4.31
Beer	80.80 ± 5.76	83.80 ± 5.43	86.00 ± 4.83	91.20 ± 3.87
*Shochu*	85.40 ± 4.24	89.20 ± 6.41	87.00 ± 3.55	86.80 ± 2.78
*Sake*	81.00 ± 11.14	82.00 ± 4.09	83.40 ± 1.40	86.40 ± 4.71
Collagen (%)				
Water	85.20 ± 5.71	95.80 ± 6.62	89.60 ± 3.98	85.20 ± 4.22
Beer	75.20 ± 11.04	84.00 ± 6.13	80.40 ± 6.88	90.20 ± 4.57
*Shochu*	86.60 ± 6.33	87.80 ± 7.68	89.00 ± 3.77	87.40 ± 3.75
*Sake*	84.20 ± 8.31	75.20 ± 7.03	81.40 ± 4.85	83.00 ± 7.69

The VAS was used to investigate the effects of alcohol beverages type on physical and emotional state. The VAS scores for tipsiness with beer, *shochu*, and *sake* at 1 and 2 h were significantly higher than that with water (*p* < 0.01, *p* < 0.05, and *p* < 0.05, respectively, at 1 h; all, *p* < 0.05 at 2 h; Table 2). However, the VAS scores for headache, palpitation, appetite, hunger, happiness, fullness, weariness, alcohol-related sickness, feeling of relaxation, and nausea were not significantly different between the beverages.

**Table 2:** Visual analogue scales scores after drinking four types of beverages in healthy subjects. Data are expressed as mean ± standard error (*n* = 5). Two-way repeated measures analysis of variance and post hoc analysis using least significant differences were used to compare clinical data between the beverages. ^∗^*p* < 0.05, ^∗∗^*p* < 0.01, compared with water.

	Before intake	After 1 h	After 2 h	After 12 h
Headache				
Water	4.4 ± 4.4	0.0 ± 0.0	0.0 ± 0.0	4.1 ± 4.1
Beer	0.0 ± 0.0	4.8 ± 4.1	1.9 ± 1.2	2.0 ± 2.0
*Shochu*	13.1 ± 13.1	11.6 ± 8.9	21.3 ± 14.8	0.0 ± 0.0
*Sake*	2.7 ± 2.1	1.6 ± 1.4	0.0 ± 0.0	0.0 ± 0.0
Palpitation				
Water	13.6 ± 5.7	11.1 ± 6.2	9.6 ± 5.9	9.1 ± 5.7
Beer	25.2 ± 11.4	41.1 ± 12.3	12.8 ± 5.3	6.1 ± 4.0
*Shochu*	10.0 ± 10.0	35.3 ± 11.2	39.2 ± 17.5	9.9 ± 7.7
*Sake*	7.2 ± 6.0	26.1 ± 16.5	18.8 ± 10.3	6.1 ± 5.3
Appetite				
Water	73.0 ± 11.7	7.9 ± 4.9	11.9 ± 6.3	37.1 ± 10.4
Beer	79.4 ± 11.1	36.4 ± 13.9	36.4 ± 13.6	38.8 ± 14.7
*Shochu*	75.5 ± 9.9	30.0 ± 16.3	7.6 ± 7.6	47.5 ± 11.6
*Sake*	71.8 ± 11.5	18.6 ± 11.0	16.9 ± 8.4	44.3 ± 13.3
Hunger				
Water	74.9 ± 11.7	8.2 ± 5.0	13.6 ± 7.6	37.4 ± 10.6
Beer	86.0 ± 7.5	36.1 ± 14.8	29.1 ± 16.6	40.5 ± 14.7
*Shochu*	61.8 ± 11.6	26.2 ± 16.7	10.2 ± 10.2	39.4 ± 14.5
*Sake*	66.7 ± 13.4	13.8 ± 9.2	16.1 ± 7.6	46.8 ± 15.4
Fullness				
Water	16.2 ± 15.2	67.1 ± 17.2	73.3 ± 13.8	15.3 ± 6.4
Beer	8.1 ± 8.1	51.2 ± 15.2	50.1 ± 10.3	6.5 ± 4.0
*Shochu*	36.8 ± 18.7	76.0 ± 9.4	71.1 ± 15.9	30.2 ± 14.9
*Sake*	17.7 ± 11.5	84.9 ± 6.7	57.8 ± 17.4	8.2 ± 4.8
Tipsiness				
Water	0.0 ± 0.0	0.0 ± 0.0	3.9 ± 3.9	0.0 ± 0.0
Beer	0.0 ± 0.0	69.2 ± 8.9^∗∗^	45.4 ± 14.6^∗^	3.6 ± 3.6
*Shochu*	0.0 ± 0.0	53.4 ± 17.3^∗^	46.0 ± 15.3^∗^	1.4 ± 1.4
*Sake*	22.3 ± 13.7	53.7 ± 17.2^∗^	40.0 ± 15.1^∗^	0.0 ± 0.0
Drunken sickness				
Water	5.9 ± 5.9	5.0 ± 5.0	3.5 ± 3.5	0.0 ± 0.0
Beer	9.7 ± 9.7	4.5 ± 2.8	8.0 ± 5.3	2.8 ± 2.8
*Shochu*	3.0 ± 3.0	29.8 ± 18.6	9.8 ± 6.0	1.4 ± 1.4
*Sake*	6.9 ± 6.3	12.7 ± 8.3	21.2 ± 19.7	0.0 ± 0.0
Weariness				
Water	18.9 ± 9.4	7.7 ± 4.9	6.5 ± 3.4	5.6 ± 5.5
Beer	11.0 ± 9.9	4.0 ± 4.0	10.0 ± 6.7	21.8 ± 8.6
*Shochu*	4.5 ± 2.9	31.6 ± 18.8	7.6 ± 5.2	13.9 ± 10.9
*Sake*	10.4 ± 10.4	13.0 ± 11.9	7.2 ± 5.4	0.0 ± 0.0
Happiness				
Water	31.8 ± 13.9	44.4 ± 12.8	47.9 ± 13.3	35.0 ± 7.9
Beer	36.3 ± 10.0	54.1 ± 7.2	54.4 ± 10.2	29.8 ± 9.0
*Shochu*	32.6 ± 12.0	39.7 ± 14.0	39.4 ± 12.2	38.0 ± 12.7
*Sake*	39.7 ± 7.5	53.0 ± 15.9	43.0 ± 14.8	46.9 ± 8.7
Relaxation				
Water	47.8 ± 16.7	55.3 ± 12.6	45.9 ± 16.9	52.7 ± 12.4
Beer	54.8 ± 16.0	66.1 ± 8.7	61.1 ± 10.5	53.3 ± 7.3
*Shochu*	55.6 ± 4.4	40.6 ± 14.3	60.9 ± 13.0	50.9 ± 9.4
*Sake*	60.9 ± 14.1	52.5 ± 15.9	49.6 ± 14.8	50.8 ± 8.5
Nausea				
Water	3.5 ± 3.5	0.0 ± 0.0	0.0 ± 0.0	0.0 ± 0.0
Beer	5.4 ± 5.4	0.0 ± 0.0	0.0 ± 0.0	0.0 ± 0.0
*Shochu*	1.8 ± 1.8	18.0 ± 13.2	8.1 ± 5.0	1.2 ± 1.2
*Sake*	0.4 ± 0.4	4.1 ± 4.1	14.2 ± 14.2	0.2 ± 0.2

In the sleep evaluation using nocturnal PSG, rapid eye movement (REM) sleep latencies with *shochu* and *sake* were significantly shorter than that with water and beer (*p* < 0.05, *p* < 0.01, respectively; Table 3). The mean total sleep time, sleep efficiency, alpha waves, and periodic movement were not significantly different between the beverages.

**Table 3:** Sleep evaluation using nocturnal polysomnography (PSG) after drinking four types of beverages in healthy subjects. Data are expressed as mean ± standard error (*n* = 5). One-way repeated measures analyses of variance were used to compare the clinical data between the beverages. ^∗^*p* < 0.05, †*p* < 0.01, compared with water and beer, respectively.

	Water	Beer	*Shochu*	*Sake*
Total PSG time (min)	720	720	720	720
Total sleep time (min)	447.2 ± 38.6	488.2 ± 17.6	482.9 ± 41.3	451.1 ± 39.6
Sleep latency (min)	151.6 ± 18.4	159.5 ± 19.3	185.3 ± 49.1	143.0 ± 10.8
REM sleep latency (min)	152.0 ± 24.6	144.2 ± 18.6	74.3 ± 6.1^∗,†^	77.9 ± 3.9^∗,†^
Stage 1 (min)	25.2 ± 5.0	37.1 ± 9.6	27.1 ± 7.4	33.9 ± 9.1
Stage 2 (min)	262.4 ± 27.8	294.2 ± 9.7	298.0 ± 33.8	270.8 ± 40.7
Stage 3 (min)	32.3 ± 6.2	34.0 ± 6.7	33.5 ± 5.6	28.2 ± 6.2
Stage 4 (min)	45.0 ± 8.3	58.0 ± 12.3	38.2 ± 7.5	34.4 ± 12.5
Stage REM (min)	82.5 ± 14.4	89.1 ± 10.2	95.1 ± 8.9	96.3 ± 13.5
Arousals index (the number per hour)	9.2 ± 1.9	13.1 ± 3.5	10.4 ± 1.6	10.3 ± 2.8
Sleep efficiency (%)	62.1 ± 5.4	67.8 ± 2.5	67.1 ± 5.7	62.7 ± 5.5
Apnea hypopnea index (the number per hour)	2.0 ± 1.0	13.7 ± 13.1	1.8 ± 0.7	1.5 ± 0.6
Snore (%)	0.1 ± 0.1	0.5 ± 0.4	1.3 ± 1.0	1.7 ± 1.6
Alpha wave (%)	0.6 ± 0.3	0.5 ± 0.3	0.6 ± 0.3	0.3 ± 0.1
Periodic limb movement (the number per hour)	3.6 ± 3.4	2.6 ± 2.5	0.1 ± 0.1	0.1 ± 0.1

**Notes.**

REM Rapid eye movement

## Discussion

In the current study, we investigated the acute effects of several types of alcohol beverages on biochemical parameters in healthy subjects. Generally, moderate alcohol consumption improves glucose tolerance and implying increased insulin sensitivity (Facchini, Chen & Reaven, 1994). Associated with it, blood glucose level affected the nutritional status at the time of consumption of alcohol (Williams, 1984). It had been reported that ethanol of the dose did not change the blood glucose concentration in healthy subject overnight fasted (Field, Williams & Mortimore, 1963; Yki-Jarvinen et al., 1998; Siler et al., 1998). Identically, blood glucose level in overnight fasted after acute alcohol intoxication was also maintained (Spolarics et al., 1994). Moreover, acute alcohol consumption did not cause hypoglycemia under the condition of adequate nutrition. In contrast, a severe and sustained hypoglycemia was elicited, when alcohol was acute ingestion to fasting 3–4 days (Field, Williams & Mortimore, 1963; Searle et al., 1974). It has been reported that long-term moderate consumption of alcohol did not alter the basal insulin concentration, however acute alcohol consumption reduced insulin secretion (Shah, Wongsurawat & Aran, 1977; Flanagan et al., 2002; Beulens et al., 2008). Ethanol reduced insulin secretion by interfering with muscarinic signaling and Protein kinase C activation in pancreatic *β*-cells (Nguyen, Lee & Nyomba, 2009), and insulin-stimulated glucose metabolism in skeletal muscle (Boden et al., 1993). Short-term exposure of alcohol inhibited the insulin-stimulated glucose transporter 4 protein translocation in skeletal myocyte in a dose dependent (Qu et al., 2011). We showed that levels of postprandial glucose and insulin differed according to alcohol beverage type. In this study, compared with water and beer, *shochu* consumption with a meal reduced the postprandial glucose levels. In addition, blood insulin levels with *shochu* were the lowest of the beverages, and the 12-h blood insulin AUC value was lower than that with beer. The insulinogenic indexes at 2 h with beer, and *sake* were higher than that with water. On the other hand, the insulinogenic index with *shochu* did not increase. These results indicate that *shochu* consumption with a meal suppresses a rise in postprandial blood glucose and insulin. Therefore, *shochu* consumption with a meal might reduce burden of the beta cell of Langerhans in the pancreas and be beneficial for the progression of type 2 diabetes compared with other alcohol beverages.

*Shochu* is a distilled beverage that does not contain carbohydrates. The differences in blood glucose and insulin levels may be explained by the carbohydrate content of the alcohol beverages (Kiechl et al., 1996; Htonen et al., 2012). One study showed that the blood glucose levels at 1 h after drinking beer or *sake* were significantly higher than that after drinking *shochu* in type 2 diabetes (Hosaka et al., 2008). In our study, the carbohydrate contents were as follows: 31.0 g in 1,000 ml of beer, 0 g in 333 ml of *shochu*, and 13.3 g in 333 ml of *sake*. On the other hand, the consumption of alcohol was equal for each of the three alcohol beverages. These findings indicate that the carbohydrate content of the alcohol beverage plays an important role in the elevation of blood glucose level after drinking.

In the beer production process in Western countries, starch is saccharified by *β*-amylase in the malt (Muralikrishna & Nirmala, 2005). *Shochu* and *sake* are produced by using *koji*, which is analogous to malt. *Koji* has been the traditional food fungus in Eastern Asia for several centuries. In the Asian brewing process, *koji* is used as a source of starch hydrolase (Yong & Wood, 1974). There are four types of *koji* according to the colors of spores or pigments produced by the cultivated molds, such as yellow *koji*, white *koji*, black *koji*, and red *koji*. In Japan, white *koji* and black *koji* have been used exclusively in production of *shochu*, while yellow *koji* has been used for production of the Japanese rice wine *sake*. In China and Korea, red yeast has been used for production of rice wine. The volatile compounds of yellow and red *koji* are distinct from those of white and black *koji*, and the different groups of volatile compounds identified in *koji* impart the respective characteristic flavors, tastes, enhanced functional content to *shochu*, rice wine, and *sake* (Heber et al., 1999; Yoshizaki et al., 2010). Many volatile compounds are also produced during the processing of beverages not only ethanol, but also other components such as n-propanol, isobuthanol, ethyl esters, and aldehydes. They are produced during fermentation by *koji* and yeast when beverages are made from materials (Yamamoto, 1961; Maarse, 1991; Yoshizaki et al., 2010).

Smells are thought to stimulate the olfactory system and produce signals that project to the olfactory bulb, where smell images are produced, analyzed and recognized by the brain (Buck, 2000). The olfactory bulb is part of the limbic system, along with the hippocampus, amygdala and hypothalamus. Olfactory stimulation is likely to have some effect on these organs (Aoshima, 2012). The hypothalamus controls the autonomic nervous, endocrine and immune system. However, the effects of aromatic compounds have not been clarified well from a scientific perspective.

Yoshizaki et al. reported that all rice *koji* had anti-obesity and anti-diabetes effects through mechanisms other than regulation of food intake. *Koji*, particularly white and red *koji*, improves glucose tolerance by increasing the expression of glucose transporter 4 protein in muscle, thereby increasing glucose uptake (Yoshizaki et al., 2014). These finding and *sake* influence glucose metabolism and contribute to suppressing the elevation of blood glucose level after drinking these alcohol beverages. However, the molecular mechanism underlying the effect of the ingredients of *shochu* and *sake* on glucose metabolism is still unclear. But the detailed mechanisms underlying these beneficial effects of *shochu* and *sake* on the glucose metabolism are not fully understood.

In the current study, there were significant differences in total ketone and 3-hydroxybutyric acid levels with *shochu* and *sake* consumption. Ketone body is a generic term for acetoacetic acid, 3-hydroxybutyric acid, and acetone. Ketone bodies are produced by the liver and used peripherally as an energy source when glucose is not readily available; their levels increase during fasting, stress, and prolonged exercise (Laffel, 1999). Alcohol diminishes hepatic gluconeogenesis and leads to decreased insulin secretion, increased lipolysis, impaired fatty acid oxidation, and subsequent ketogenesis (Lefèvre, Adler & Lieber, 1970). In our studies, these differences might be caused by the amount of activity or stress levels of the subjects because the experiments were performed at night to enable simultaneous measurement of the sleep changes using polysomnography. Blood lactic acid levels also increased 2 h after drinking beer, *shochu*, and *sake*. It has been reported that alcohol inhibits lactate-stimulated gluconeogenesis when given acutely (Kreisberg, Owen & Siegal, 1971). Additionaly, it increases the lactate output from skeletal muscle (Jorfeldt & Juhlin-Dannfelt, 1978).

Ethanol affects many functions of the brain, tissues and peripheral organs. Ethanol acts on the central nervous system through the action, such as enhancement of the Ion channel *γ*—aminobutyric acid type A (GABAA) receptors (Wafford et al., 1990), Inhibition of N-methyl-D-aspartate (NMDA) receptors (Lovinger, White & Weight, 1990), and opening of the G-protein—activated inwardly rectifying K+ channels (Lewohl et al., 1999). Thereby affected the drunkenness, mental function, and sleep conditions. Harburg et al. (1993) reported that intoxication is affected by age, sex, ethanol level, and psychosocial factors. In addition, Scholey et al. (2012) reported that there were highly significant correlations between measured blood alcohol concentrations and sober-drunk VAS scores. In the present study, the feeling of tipsiness lasted longer after drinking beer, *shochu*, or *sake* than that with water. However, there was no correlation between postprandial alcohol concentration and tipsiness scores. The rate of change in blood alcohol concentrations and other factors may influence the duration of the feeling of tipsiness.

In the present study, the effects of alcohol beverages on sleep were investigated. REM sleep latency after drinking *shochu* was significantly different compared with the other beverages. REM sleep latency is the duration from sleep onset to the onset of the first REM sleep. Previous studies have shown that REM sleep latency decreases with several psychiatric disorders, including schizophrenia, narcolepsy, and borderline personality disorder (Reynolds et al., 1985; Rotenberg et al., 2002). In contrast, exercise increases REM sleep latency (Driver & Taylor, 2000). Recently, it has been reported that administration of escitalopram, a selective serotonin reuptake inhibitor, increases REM sleep latency (Vas et al., 2013). It remains to be determined whether drinking alcohol beverages chronically influences sleep patterns and psychological function.

## Conclusions

Our results suggest that acute consumption of alcohol beverages with a meal results in different responses in biochemical parameters, physical and emotional state, and sleep patterns depending on the type of alcohol. The traditional Japanese alcohol beverages, especially *shochu*, showed an inhibitory effect on increased postprandial glucose and insulin levels while maintaining the feeling of tipsiness. The differences by alcohol type could be important for better control of diseases related to insulin resistance, such as metabolic syndrome.

## Supplemental Information

10.7717/peerj.1853/supp-1 Supplemental Information 1 Raw data

10.7717/peerj.1853/supp-2 Supplemental Information 2 Raw data

## Additional Information and Declarations

### Data Availability

The following information was supplied regarding data availability: Raw data has been supplied as Supplemental Dataset files.
